# Reliability and validity of PedsQL for Portuguese children aged 5–7 and 8–12 years

**DOI:** 10.1186/s12955-014-0122-3

**Published:** 2014-09-11

**Authors:** Pedro L Ferreira, Carla F Baltazar, Luís Cavalheiro, Jan Cabri, Rui S Gonçalves

**Affiliations:** University of Coimbra, Centre for Health Studies and Research, Coimbra, Portugal; University of Coimbra, Faculty of Economics, Coimbra, Portugal; Dona Estefânia Hospital, Lisbon, Portugal; Polytechnic Institute of Coimbra, Coimbra Health School, Coimbra, Portugal; Norwegian School of Sport Science, Department of Physical Performance, Oslo, Norway

**Keywords:** PedsQL, Children, Chronic illness, Health-related quality of life, Pediatrics

## Abstract

**Background:**

Pediatric Quality of Life Inventory (PedsQL) is a measure to assess health-related quality of life (HRQoL) in children and adolescents. It is formed by 23 items adapted to children age and includes a parent proxy report version. With four multidimensional subscales and three summary scores, it measures health as defined by WHO. The concepts measured by this instrument are ‘physical functioning’ (8 items), ‘emotional functioning’ (5 items), ‘social functioning’ (5 items) and ‘school functioning’ (5 items). It also measures a ‘total scale score’ (23 items), a ‘physical health summary score’ (8 items) and a ‘psychosocial health summary score’ (15 items). The aim of this paper is to present the main results of the cultural adaptation and validation of the PedsQL into European Portuguese.

**Methods:**

The Portuguese version was the result of a forward-backward translation process, with a cognitive debriefing analysis, guaranteeing face validity and semantic equivalence. Children aged 5–7 and 8–12 were randomly selected and were asked to fill a socio-demographic data survey and the Portuguese versions of PedsQL and KINDL, another HRQoL measure for children and adolescents. They were divided into three groups, healthy children, children with type I diabetes and children with spina bifida.

The reliability was tested for reproducibility (ICC) and internal consistency (Cronbach’s alpha). The construct validity (known-groups discriminant validity) was supported by differences between self-reports from healthy children and children with chronic conditions, and from children with chronic diseases and their parents. The criterion validity was tested after the correlations of the scores obtained by both children and adolescents HRQoL assessment instruments.

**Results:**

A total of 179 children and 97 parents were recruited. PedsQL demonstrated good levels of reproducibility (*r* > 0.95 in all versions) and acceptable levels of internal consistency with Cronbach’s alpha at 0.70 on most scales. Concordance values between children’s and parents’ perceptions ranged between 0.36 and 0.78 and the correlations with KINDL questionnaire were excellent, supporting concurrent validity.

**Conclusions:**

The Portuguese version of the PedsQL demonstrated acceptable psychometric properties for future research and clinical practice for children aged 5–12.

## Introduction

With the declining prevalence of acute childhood diseases, the treatment and management of chronic conditions currently constitutes a large proportion of the work performed in the daily medical routine. These conditions last for many years and represent a considerable personal and financial burden for children and their parents [1]. Regular stays in hospitals, painful treatments and an uncertainty about the future may compromise both the child**’**s and their family’s quality of life [2].

Children, in particular those needing special healthcare, are more vulnerable to changes and face various challenges which have an impact on their health-related quality of life (HRQoL) [3]. It is argued that the HRQoL instruments designed to measure and monitor healthcare in children with chronic conditions should be available, accessible and easy to use [4,5], in order to facilitate the assessment of the impact of the disease and treatments, to support daily practice and clinical research [6,7].

After the success reached by the design and the use of several HRQoL instruments for adults, a number of generic and disease-specific measures have been developed for use in pediatrics. Their availability makes it possible to routinely measure children’s functioning, without imposing long and inadequate questionnaires on them or their parents [8]. Examples of these tools are the KINDL Questionnaire [9], the Child Health Questionnaire (CHQ) [10] and the Pediatric Quality of Life Inventory (PedsQL) [11]. All these instruments aim at measuring the subjective perception of disease and treatment impact on the children’s physical, psychological and social functioning, taking into account their age and their expected activities.

The PedsQL 4.0 Generic Core Scales is an instrument designed to measure the generic HRQoL in children, adolescents and their parents. It is composed by 23 items that measure physical, emotional, social and school functioning, and has been demonstrated to be psychometrically valid and reliable [12,13]. There are seven versions, taking into account the respondent’s age and source of information. For children, the following versions are available: 5–7 years of age (interview); 8–12 years, and 13–18 years. For parents, 2–4, 5–7, 8–12 and 13–18 age groups versions are available.

Items are scored on a 5-point Likert scale for children and adolescents aged 8–18 and for all the parents’ versions. In this scale, zero (0) means never, and four (4) means almost always. In the 5–7 children versions, the faces scale uses the anchors 0 (never), 2 (often) and 4 (very often). Items are inversely scored and linearly transformed from zero (0) to one hundred (100) (0 = 100, 1 = 75, 2 = 50, 3 = 25 and 4 = 0). Scores can be obtained for each of the measured scales or grouped into two major dimensions: physical health (physical functioning items), and psychosocial health (emotional, social and school functioning items) or in a total score, resulting from the sum of all items, divided by the number of the items with a valid answer. Higher scores point to a better HRQoL. If more than 50% of items are missing for a scale, the score is not given [12,13].

The purpose of this paper was to report on initial reliability and validity of the Portuguese versions of the PedsQL 4.0 Generic Core Scales for ages 5–7 and 8–12 years in healthy and children with chronic illness.

## Methods

### Cultural Adaptation

After author’s consent, the cultural adaptation of PedsQL, for each age group 5–7 and 8–12 years, followed the sequential approach [14], as recommended by Mapi Research Institute [15]. Two Portuguese translators, fluent in English were selected to, independently; perform the English/Portuguese translations, which have been merged during a consensus meeting. This version was then sent to two English native bilingual translators who performed the corresponding back-translations, which were also compared among each other and to the original version. Afterwards, the amended Portuguese version was given to a panel of experts composed by two physical therapists, a physician and a health outcomes expert to analyze the equivalence of meanings of the translated items.

Next, to face validate the Portuguese versions of PedsQL and to study how understandable and acceptable the measures were when implemented, the Portuguese versions were tested by two panels of twelve children and their parents, for each age group. The Portuguese children/parents 5–7 and 8–12 years versions of PedsQL resulted from the consensus obtained from this procedure.

### Instruments

Besides the Portuguese versions of the PedsQL, the validation protocol included the following measures:

- ◾ The KINDL questionnaire, another generic health measure for children composed by the “Kiddy” version for parents with children between the ages of 4–7 years, and by the “Kid” versions for parents and children 8–12 years of age. Each of these tools contains 24 items to measure the physical well-being, emotional well-being, self-esteem, family, friends and school dimensions. The scores are expressed by subscales and by a total score, obtained by summing the individual scores, transformed into a 0–100 scale, in which higher values mean a better health status [9,16];
- ◾ Children’s socio-demographic and clinical information, including gender, age and clinical condition;
- ◾ Parents’ socio-demographic and clinical information, including the degree of kinship, gender, age, education level and marital status.

The paper-based questionnaires were self-administered to both parents and children aged between 8 and 12, and administered by interviews to children aged between 5 and 7 years old. When applied in hospital environment, the questionnaires were filled before medical appointments, and children filled them in without being accompanied by their parents.

### Reliability

The reliability tests were conceived in terms of reproducibility and internal consistency. The reproducibility study in the 5–7 age group was concluded by using a subsample of six diabetic and six spina bifida children as well as 10 parents. Regarding the 8–12 age group, seven diabetic and five spina bifida children and 10 parents participated in the test. For all subsamples the 72-hours intraclass correlation coefficient (ICC) were computed by formula 2,1 [17]. The same coefficient was chosen for the item-to-item reproducibility study. A reproducibility coefficient above or equal to 0.70 was considered acceptable [17]. The time interval was chosen in such a way that the respondents would not remember previous answers and also so that it would not be likely for a real change on health to occur.

The internal consistency was assessed by the Cronbach’s alpha in a sample of children and parents. Values between 0.70 and 0.95 were considered acceptable reliability indicators [17].

### Validity

To test the construct validity and the criterion validity we used a sample of healthy children recruited with the collaboration of basic schools at Oeiras Municipal Council, and a sample of diabetic and spina bifida children recruited at the Pediatric Endocrinology and Neurology outpatient consultations from the Lisbon Pediatric Hospital, as well as the corresponding parents of the children with chronic illness. Children with a reasonable understanding level were asked to fill the questionnaire with a prior consent of their parents. Children with acute symptomatology were excluded. Another criterion for exclusion was the existence of cognitive problems on parents which would impede the evaluation of their child’s health status.

Known-groups discriminant validity was evaluated through the analysis of scores obtained by healthy and chronically ill children. Using the MANOVA for the four PedsQL subscales, we looked at this effect. Four test statistics were computed using the eigenvalues of the model: the Pillai’s Trace, the Lawley-Hotelling’s Trace, the Wilk’s Lambda and the Roy’s Largest Root. Student’s *t*-test was also used for total scores. Analyses of the relations between the scores obtained by simultaneously applying PedsQL and KINDL questionnaires were performed as well. The relations were studied by using the Pearson’s correlation coefficient. Cohen and Holiday criteria [18] were applied to interpret these correlation coefficients, suggesting the following categorization: very low correlation for values below 0.20; low correlation for values between 0.20 and 0.39; moderate correlation for values between 0.40 and 0.69; high correlation for values between 0.70 and 0.89 and very high correlation for values above 0.89.

### Statistical Analysis

The level of confidence was chosen to be at 95% (*p* < 0.05). SPSS® version 22 for Windows® was used for the statistical analysis.

### Ethics

The study was approved by the ethics committee of the Lisbon Pediatric Hospital.

## Results

### Cross-cultural Adaptation

Following the opinion of parents and children participating in the panels, PedsQL is seen as a short questionnaire, quick and easy to answer and to understand, useful and suitable for the target population. It was also consensual the fact that the language was simple, clear and colloquial. It took between 3 and 10 minutes to answer for children between 5–7 years old, and 1 to 7 minutes for their parents. For the 8–12 years version, children took between 3 and 6 minutes to answer and the parents took between 2 and 5 minutes.

The most problematic concepts were “household chores” and “school work”, but only for some 5 year old children. Few children, at that age, help their parents around the house and not all of them go to school. However, applying the measure through interviews for this age group, easily overcomes some of the difficulties inherent to understanding the concepts in question.

No other problematic questions or concepts were identified by panels, and no other difficulties were mentioned in relation to the content of other translated items. The final versions were therefore prepared and sent to the measure’s original author, who gave us his formal permission for the Portuguese versions of PedsQL 4.0, for the 5–7 and 8–12 age groups.

### Sample

For the validity study the Portuguese versions of PedsQL were applied to a sample of 179 children and 97 parents, whose distribution is given in Table 1.

**Table 1:** **Distribution of the subjects selected for the validity study**

	**5-7 age group**			**8-12 age group**			**Total**
	**Children**		**Parents**	**Children**		**Parents**	
	**M**	**F**		**M**	**F**		
Healthy	16	16	------	25	25	------	82
Diabetes	8	10	18	17	13	30	96
Spina bifida	7	11	18	16	15	31	98
Total	31	37	36	58	53	61	276

83.3% of parents were female (78.1% mothers), married and with a mean age of 38 years. Regarding the education level these parents were uniformly distributed across all levels.

Table 2 presents the main distribution indicators for the total scale score and for each PedsQL dimension.

**Table 2:** **Distribution of descriptive for PedsQL child reports and parent-reports**

	**PedsQL scores**	**5-7 age group**		**8-12 age group**	
		***mean***	***s.d.***	***mean***	***s.d.***
Children reports	Total scale score	75.45	12.04	75.74	14.27
	Physical functioning	77.39	18.42	78.04	19.55
	Psychosocial health	74.41	10.99	74.52	13.81
	Emotional functioning	74.71	15.88	70.05	18.25
	Social functioning	76.32	14.85	80.72	17.84
	School functioning	72.21	17.69	72.79	15.16
Parents-reports	Total scale score	68.78	17.28	69.19	15.48
	Physical functioning	68.40	21.40	68.90	23.02
	Psychosocial health	68.98	16.88	69.34	14.07
	Emotional functioning	65.83	16.97	65.82	19.24
	Social functioning	75.42	21.92	76.23	20.01
	School functioning	65.69	21.32	65.98	16.04

### Reliability

Table 3 contains Cronbach’s alphas and the intraclass correlation coefficient for the European Portuguese versions of PedsQL for each of the subscales and for the total score of each version. In relation to the reproducibility of each item, values generally were 0.8 or higher. However, 5–7 children showed lower internal consistency than any other sample.

**Table 3:** PedsQL internal consistency and test- retest reliability

**PedsQL dimensions**	**Children 5-7**		**Parents of children 5-7**		**Children 8-12**		**Parents of children 8-12**	
	***α***	ICC*	***α***	ICC*	***α***	ICC*	***α***	ICC*
Total scale score	0.78	0.97 (0.88-0.99)	0.92	0.99 (0.95-0.99)	0.89	0.98 (0.95-0.99)	0.89	0.99 (0.97-0.99)
Physical functioning	0.73	0.95 (0.83-0.97)	0.86	0.97 (0.90-0.99)	0.84	0.98 (0.94-0.99)	0.86	0.99 (0.85-0.99)
Psychosocial health	0.60	0.94 (0.76-0.99)	0.88	0.98 (0.92-0.99)	0.83	0.95 (0.85-0.99)	0.82	0.99 (0.95-0.99)
Emotional functioning	0.41	0.83 (0.26-096)	0.74	0.98 (0.90-0.99)	0.74	0.95 (0.86-0.99)	0.79	0.90 (0.66-0.98)
Social functioning	0.36	0.80 (0.46-0.94)	0.80	0.98 (0.93-0.99)	0.71	0.93 (0.77-0.99)	0.75	0.92 (0.73-0.98)
School functioning	0.63	0.98 (0.91-0.99)	0.81	0.98 (0.91-0.99)	0.62	0.94 (0.80-0.98)	0.64	0.98 (0.91-0.99)

*ICC – Intraclass correlation coefficient (lower bound-upper bound).

### Validity

As expected, healthy children evaluated their health status more positively than children with chronic health conditions. These differences can be noted in Table 4 by looking at the univariate total scale score for both age groups (*p* < 0.0001). This table also provides the means and the standard deviations for the two different factors (healthy children and children with chronic illness).

**Table 4:** **PedsQL™ self-report comparing healthy and ill children**

		**5-7 age group**				**8-12 age group**			
		**Healthy children (n=32)**		**Children with chronic illness (n=36)**		**Healthy children (n=50)**		**Children with chronic illness (n=61)**	
		***mean***	***s.d.***	***mean***	***s.d.***	***mean***	***s.d.***	***mean***	***s.d.***
**Total scale score**		**80.91**	**6.69**	**70.59**	**13.66**	**82.37**	**8.57**	**70.31**	**15.70**
		**t (66) = 3.88;** ***p*** **<0.0005**				**t (109) = 4.86;** ***p*** **<0.0005**			
PedsQL subscales	Physical functioning	86.72	9.75	69.10	20.37	86.38	11.20	71.21	22.21
	Emotional functioning	80.00	14.81	70.00	15.49	73.20	13.58	67.46	21.09
	Social functioning	77.19	11.43	75.56	17.48	89.00	12.45	73.93	18.78
	School functioning	76.25	10.70	68.61	21.67	78.50	12.51	68.11	15.63
		Pillai’s Trace = 0.281; *p*<0.0001				Pillai’s Trace = 0.213; *p*<0.0001			
		Hotelling’s Trace = 0.392; *p*<0.0001				Hotelling’s Trace = 0.270; *p*<0.0001			
		Wilk’s Lambda = 0.718; *p*<0.0001				Wilk’s Lambda = 0.787; *p*<0.0001			
		Roy’s Largest Root= 0.281; *p*<0.0001				Roy’s Largest Root= 0.212; *p*<0.0001			
Tests of between-subjets effects	Physical functioning	F (1,66) = 19.87; *p*<0.0001				F (1,109)=19.27; p<0.0001			
	Emotional functioning	F (1,66) = 7.36; *p*<0.009				F (1,109)=2.79; p<0.099			
	Social functioning	F (1,66) = 0.20; *p*<0.655				F (1,109)=23.64; p<0.001			
	School functioning	F (1,66) = 3.26; *p*<0.075				F (1,109)=14.47; p<0.001			

*s.d.* – standard deviation.

Regarding the MANOVA test statistics and the observed *p*-values associated we can reject the hypothesis that the given predictor (having or not a chronic disease) has no effect on the four PedsQL subscales. On the other hand, analyzing the univariate tests of between-subjects effects we may evidence that, for the 5–7 group, chronic illness has a statistically significant effect on physical and emotional functioning. Regarding the 8–12 age group, all subscales, except emotional functioning, showed to be associated to statistically significant effects.

Moreover, according to parents (data not shown in this paper) the different impact in health status of diabetes versus spina bifida for children aged between 5 and 12 was only significant for older children, that is, for the 8–12 age group.

Table 5 shows the values of the relations between scores registered by self-evaluation of ill children (spina bífida and diabetes) and their parents’ perceptions. The only subscale where no significant value association was observed was emotional functioning for the 5–7 age group.

**Table 5:** **PedsQL – Correlations between parent-reports and children with illness self-report**

**PedsQL dimensions**	**Total sample**	**Children 5-7**	**Children 8-12**
Total scale score	0.64**	0.41*	0.78**
Physical functioning	0.67**	0.50**	0.76**
Psychosocial health	0.59**	0.36*	0.74**
Emotional functioning	0.57**	0.26	0.69**
Social functioning	0.61**	0.48**	0.70**
School functioning	0.66**	0.66**	0.66**

*Correlation is significant at *p* < 0.05; **Correlation is significant at *p* < 0.01.

The associations registered by simultaneously applying both generic health status measures PedsQL and KINDL for the 5–7 version (parents evaluation) and 8–12 years of age version for children and their parents respectively, are shown in Table 6.

**Table 6:** **PedsQL vs. KINDL, children and parents reports**

	**PedsQL**	**KINDL**						
		**Physical well being**	**Emotional well being**	**Self-esteem**	**Family**	**Friends**	**School**	**KINDL total**
Parent of children 5-7	Total scale score	0.54**	0.50**	0.42*	0.12	0.34*	0.50**	0.56**
	Physical functioning	0.41*	0.38*	0.47**	0.10	0.30	0.44**	0.49**
	Psychosocial health	0.58**	0.53**	0.34*	0.13	0.33	0.49**	0.55**
	Emotional functioning	0.70**	0.56**	0.25	0.19	0.33	0.44**	0.56**
	Social functioning	0.36*	0.45**	0.28	0.15	0.46**	0.41*	0.48**
	School functioning	0.45**	0.35*	0.33*	−0.00	0.05	0.38*	0.37*
Parents of children 8-12	Total scale score	0.48**	0.63**	0.14	0.26*	0.46**	0.35**	0.64**
	Physical functioning	0.35**	0.49**	0.13	0.29*	0.46**	0.22	0.53**
	Psychosocial health	0.50**	0.62**	0.13	0.19	0.37**	0.40**	0.61**
	Emotional functioning	0.51**	0.61**	0.11	0.21	0.18	0.18	0.50**
	Social functioning	0.30*	0.35**	0.06	0.19	0.49**	0.36**	0.48**
	School functioning	0.32*	0.46**	0.14	0.01	0.14	0.38**	0.41**
Children 8-12	Total scale score	0.45**	0.59**	0.03	0.15	0.45**	0.30**	0.51**
	Physical functioning	0.34**	0.38**	0.02	0.11	0.29**	0.15	0.33**
	Psychosocial health	0.46**	0.65**	0.03	0.16	0.49**	0.36**	0.56**
	Emotional functioning	0.31**	0.52**	0.03	0.05	0.18	0.21*	0.34**
	Social functioning	0.40**	0.54**	0.03	0.16	0.64**	0.27**	0.52**
	School functioning	0.42**	0.52**	0.02	0.19*	0.38**	0.42**	0.49**

*Correlation is significant at *p* < 0.05; **Correlation is significant at *p* < 0.01.

For all types of respondents and age groups, all correlations are positive and the majority is of low or moderate magnitude.

## Discussion

In this paper we presented the process of cross-cultural adaptation of the European Portuguese translation of the PedsQL 4.0 Generic Core Scales for the ages 5–7 years and 8–12 years, and provided initial evidence of its reliability and validity in a sample of healthy children, children with type I diabetes and spina bifida, as well as their parents.

All translations were done in direct collaboration with Mapi, with the author and the Center for Health Studies and Research of the University of Coimbra (CEISUC). Other languages have also followed the same methodology, including German [19], UK English [20], Russian [6], Greek [21], Argentinean Spanish [22] and Brazilian Portuguese [23] languages. For the adopted methodology and the results obtained, it can be said that the Portuguese versions of PedsQL 4.0 are semantically equivalent, meaning that PedsQL items were also valid for the Portuguese context. The use of lay people for the panels is quite an usual mode for the content validity analysis of a measuring instrument [12]. Parents and children who took part in the panels considered that the measures had no problems in terms of clarity and acceptance. Thus, the results suggest that the Portuguese versions of PedsQL4.0 have an acceptable level of content validity. Also, comparing reliability and validity scores obtained by this study with those of the original version, suggests the potential use of the Portuguese version for routine clinical practice and research in Portugal [13,14,19-27].

ICC is the most appropriate and widely used indicator to test the reproducibility of continuous measures. The results of our study, always above or equal to 0.80, prove that the adapted versions showed good levels of reproducibility, for the dimensions and PedsQL total.

Cronbach’s alpha for the Portuguese version of PedsQL and its main dimensions (physical functioning and psychosocial health) confirm the good levels of internal consistency. Only the psychosocial dimension for the 5–7 children version showed a score below 0.70, as in other studies [22,25,26]. In these studies and in the study of Bastiaansen et al. [28], the alphas obtained for the subscales emotional, social and school functioning by children aged 5–7 years also had a consistent tendency to be low.

Even though the values obtained in the 5–7 children version were below 0.70 in the emotional, social and school functioning subscales, it is recognized that in this version these subscales can be used for a descriptive and exploratory analysis of specific function domains [14]. In this age group, the perception of time and the difficulty in understanding the disease, may affect the reliability of their own health assessment and may explain these findings. In fact, due to the cognitive developmental level of young children, we may expect some difficulties in completely understanding health concepts and, consequently, they show some difficulties in assessing their own health status [29].

However, child versions for the 5–7 and 8–12 age groups of PedsQL 4.0 clearly showed the ability to discriminate between children with chronic conditions and healthy children. In fact, the differences in scores in almost all dimensions and subscales of this measure demonstrate so. Likewise, in the English version’s validity study, children with chronic illness generally had lower scores than healthy ones [20]. Studies on children suffering from spina bifida also showed lower scores for each dimension measured by PedsQL 4.0 than healthy children [11].

In the 5–7 and 8–12 parent versions comparisons were only drawn between groups of children with distinct disorders - diabetes versus spina bifida – and the scores obtained only discriminate for the 8–12 age group. Although spina bifida is recognized as a condition that causes greater disability than diabetes, it is natural that the distinction between the adverse impacts of either conditions becomes more evident as children get older. The study of Varni and Rode also reported significantly lower values for parents of children with spina bifida than for parents of children with other chronic disorders [11].

There is generally good agreement in our sample between children’s perceptions in what concerns their health status and the views of their parents. This agreement is greater for the physical than for the psychosocial domain. Several studies tally with ours in observing both the strength of the relations and the fact that they are higher for the physical dimension than for the emotional and social dimensions of health state [14,23,26,27,30] Eiser’s systematic review [31] reports values for good agreement (*r* ≥ 0.50) between the assessment of the children and that of their parents in the domains that reflect physical activity, functioning and certain symptoms, but weak agreement (*r* < 0.30) for the emotional and social domains of HRQoL. It is also assumed that there is a better agreement for more easily observed behaviors associated with the functional state, and a poorer for cognitive or emotional qualities like fear and anxiety. The results, as those from some other studies already cited, with a predominance of low to moderate value relations, only confirm that there is a need to measure both perspectives, because the perception of children and the views of parents complement one another when pediatric HRQL is assessed in the clinic or in research [1,31].

When we related PedsQL 4.0 to KINDL we found, as expected, low to moderate associations between the two measures. All the PedsQL scores, in all versions considered, were systematically associated with the total KINDL, which supports the validity of PedsQL. Although both are children’s health status tools, they do not necessarily measure the same constructs, nor generate the same health profiles, and so, the value of the relations would not necessarily be higher. For example, someone might expect a higher correlation value between the physical performance dimensions of PedsQL and the physical well-being of KINDL. But, while in the former, the gathering of information focuses more on the ability to carry out physical activities (six of the eight items on the list), in the latter, the items are more concerned with physical symptomatology which can engender illness (e.g. having headache or stomach ache, feeling strong and full of energy).

As a rule, PedsQL dimensions do not correlate with the self-esteem and family subscales of KINDL. The constructs measured by KINDL are either not present in PedsQL or are dispersed in some of its subscales. Having pride in oneself, feeling the greatest or getting on with one’s parents, feeling good at home are items in the self-esteem and family dimensions of KINDL for which there are no direct equivalents in any of the PedsQL dimensions under consideration.

## Conclusions

This study was constrained by the fact that the perception of parents about their children’s health status was not assessed and the fact that the subsample of children with chronic conditions was drawn from a single hospital. Having access to the view of the parents of the healthy children and working with a greater sample number of children with an illness could well strengthen our findings.

In conclusion, according to our results, the European Portuguese versions of PedsQL 4.0 Generic Core Scales, age groups 5–7 and 8–12, for children and their parents, are semantically equivalent to the original measure, they offer good levels of reliability and acceptable levels of validity; their use is recommended for clinical practice and research in Portugal.
